# Incidental Cold Agglutinins in Lung Transplant Recipients

**DOI:** 10.1097/TXD.0000000000001795

**Published:** 2025-04-17

**Authors:** Isabelle Moneke, Axel Semmelmann, David Schibilsky, Torsten Loop, Elke Weinig, Ömer Senbaklavaci, Johannes Kalbhenn, Florian Emmerich

**Affiliations:** 1 Department of Thoracic Surgery, Medical Center—University of Freiburg, Faculty of Medicine, University of Freiburg, Freiburg, Germany.; 2 Department of Cardiac Surgery, University Heart Center—Freiburg Bad Krozingen, Faculty of Medicine, University of Freiburg, Freiburg, Germany.; 3 Department of Anaesthesia and Critical Care Medicine, Medical Center—University of Freiburg, Faculty of Medicine, University of Freiburg, Freiburg, Germany.; 4 Institute for Transfusion Medicine and Gene Therapy, Medical Center—University of Freiburg, Faculty of Medicine, University of Freiburg, Freiburg, Germany.; 5 Department of Thoracic Surgery, Faculty of Medicine, University of Zurich, Zurich, Switzerland.

## Abstract

**Background.:**

The relevance of cold agglutinins in lung transplantation (LTx) recipients is unclear. While there is typically no intentionally induced hypothermia, the cold preservation of organs could potentially lead to microvascular injury and vascular occlusion after implantation and reperfusion in the presence of cold agglutinins. This study aims to analyze the impact of cold agglutinins in lung transplant recipients on short- and long-term outcomes after LTx.

**Methods.:**

We retrospectively analyzed the medical records of 251 patients who underwent LTx at our institution between March 2003 and June 2023. One hundred seventy-three patients were included in the study. Statistical analysis was performed using SPSS and GraphPad software.

**Results.:**

One hundred seventy-three of 251 (69%) of the lung transplant recipients were tested for cold agglutinins, which were positive in 78 of 173 (45%) patients. Most had a temperature amplitude of 4 °C; a broader temperature amplitude was detected in 9 of 78 (12%) patients. While there was no effect on overall long-term survival, cold agglutinins were associated with an increased incidence of reperfusion edema (*P* = 0.0002), severe primary graft dysfunction grade 2/3 (PGD2/3; *P* = 0.001), and early postoperative thromboembolism (*P* = 0.04). Multivariate analysis revealed PGD2/3 and thromboembolism as independent predictors of reduced long-term survival (*P* = 0.003 and *P* = 0.003, respectively). Plasmapheresis shortly before LTx in selected patients with a high cold agglutinin titer and broad temperature amplitude removed the cold agglutinins for at least 2 mo with good patient outcomes.

**Conclusions.:**

Cold agglutinins are associated with an increased incidence of reperfusion edema, PGD2/3, and early postoperative thromboembolism after LTx. Further studies are warranted to evaluate the benefits of regular screening.

## Graphical abstract

**Figure:**
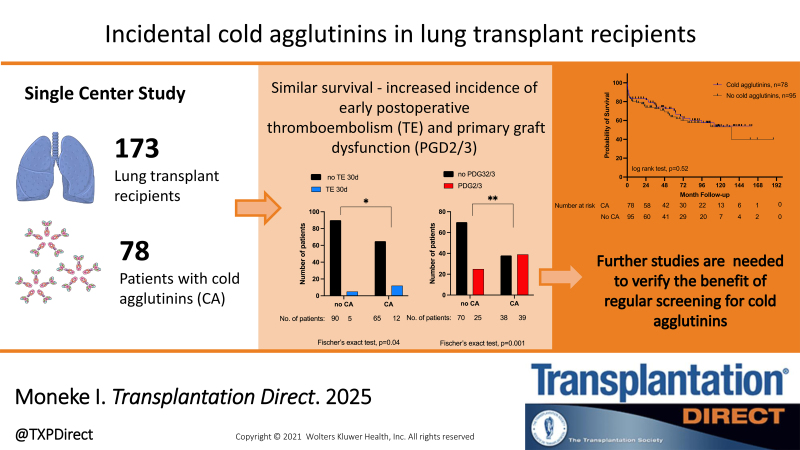

Lung transplantation (LTx) is currently the most effective treatment option for selected patients with end-stage chronic pulmonary disease.^1^ The immediate postoperative phase is often crucial, and prevention and detection of early postoperative complications can improve short- and long-term outcomes after transplantation.^2^

Cold agglutinins (CAs) are IgM red blood cell autoantibodies that preferentially bind to erythrocytes at lower-than-core body temperature and can cause erythrocyte agglutination because of their multivalent structure.^3^ They activate at varying levels of hypothermia and are rarely of clinical significance at normothermia.^4^ Primary CA disease (CAD) accounts for about 15% of autoimmune hemolytic anemias.^5^ It is associated with a low-grade clonal B-cell lymphoproliferative disorder^6^ and usually presents with anemia, acrocyanosis, and fatigue.^3^ Moreover, patients with CAD have an increased incidence of thromboembolism.^6-8^ CAs can also develop secondary to other diagnoses, such as infection with *Mycoplasma pneumoniae*, Epstein-Barr virus, aggressive lymphoma,^5^ or fibrotic lung disease.^9,10^ The term “cold agglutinin syndrome” is commonly used to describe this more heterogeneous and less common form.^6^

In patients undergoing cardiovascular operations requiring intentionally induced hypothermia, CAs can cause severe complications, for example, hemolysis, renal failure, myocardial infarction, or cerebral damage.^11^ Currently, data regarding the importance of routine preoperative screening are controversial, and there is no consensus regarding managing these patients.^12-14^

Guidelines for managing patients with asymptomatic CAs detected in routine hematological screening before cardiac surgery have been suggested, but they are based on small studies.^15^ CAs bind to their antigen at an optimum temperature of 3–4 °C. However, they can react at a higher temperature, depending on their thermal amplitude, which is defined as the highest temperature at which the CA will bind to its antigen.^16^ Patients with low CA titer (1:32) may not be at risk of agglutination during surgery, but in patients with high titers, preoperative strategies (eg, steroids, cyclophosphamide, IgG, or plasma exchange therapy) have been suggested to reduce antibody titer or reactivity,^17^ especially in case of a clinically relevant temperature amplitude.

In solid organ transplantation, CAs have been associated with allograft dysfunction.^18,19^ While there is limited data regarding kidney and liver transplantation, there is almost no data regarding CAs in LTx. The main reason for this might be that hypothermia is not necessary for lung transplant surgery per se; however, because of cold preservation of the organs to mitigate the deleterious effects of ischemia, activation of CAs could occur after implantation and reperfusion of incompletely rewarmed lungs and potentially lead to microvascular injury and thrombosis.^20^

This study aims to analyze the incidence of CA detection in our lung transplant recipient cohort, explore its relevance regarding perioperative complications, and whether regular screening might be beneficial before transplantation.

## MATERIALS AND METHODS

### Design and Study Population

We performed a retrospective single-center analysis with 251 patients (133 males and 118 females) who underwent LTx at the Department of Thoracic Surgery, Medical Centre—University of Freiburg between March 2003 and June 2023. Recipients of combined transplantations, such as heart transplantation/LTx, were excluded. One hundred seventy-three patients were routinely tested for CAs before transplantation, according to the current in-house guidelines by our Department of Transfusion Medicine and Gene Therapy and included in the study. One patient underwent preoperative plasmapheresis during the study period and was excluded from further analysis. All patients were asymptomatic before screening; no patient had a history of symptomatic CAD. Data were collected by analyzing electronic medical records and discharge and autopsy reports. Laboratory test results, lung function analysis, and bronchoscopic biopsies were collected in regular clinical follow-ups at our transplant outpatient center.

### Definitions

Pulmonary edema was defined based on the presence of parenchymal infiltrates in the allograft on the immediate postsurgical chest radiograph in the absence of other etiologies for example pneumonia or cardiac pulmonary edema.

Primary graft dysfunction was defined according to the International Society for Heart and Lung Transplantation criteria based on the presence of diffuse parenchymal infiltrates in the allograft on the chest radiograph within 72 h and the Pao_2_/FiO_2_ ratio.^21,22^ A radiologist and a transplant clinician evaluated all X-rays, and Pao_2_/FiO_2_ ratios were taken from the intensive care unit (ICU) files. Primary graft dysfunction grade 2/3 (PGD2/3) at 72-h post-LTx was considered clinically relevant.

The body mass index (BMI) was used to categorize patients as underweight (BMI < 18.5), of normal weight (18.5–24.9), overweight (25.0–29.9), or obese (BMI > 30.0).

Early postoperative thromboembolism was defined as thromboembolism occurring within 30 d of LTx. Patient files (including the respective ICU and intermediate care unit [IMC] records) were checked to get an accurate count of events.

### Lung Preservation

Since 2020, PERFADEX Plus solution (XVIVO) has been used for lung preservation. Before 2020, PERFADEX supplemented with tris-hydroxymethyl aminomethane and calcium ions was used. After cold preservation, the lungs were stored in a cooler filled with ice for transport. This usually leads to arrival at the recipient hospital at a temperature of 0–4 °C.^23^ After implantation vascular clamps are released gradually for reperfusion of the transplanted lung.

### Testing for CAs

CAs were measured by agglutination test in tubes at 3 different temperatures (4 °C, 22 °C, 37 °C) using cord cells, 0 cells, and auto controls. Direct antiglobulin testing was performed by column agglutination according to the manufacturer’s recommendations (ID-DiaPanel, Coombs Anti IgG; Bio-Rad Laboratories).

### ICU Management

Physical therapy was available to all patients starting from the first postoperative day in the ICU. Additionally, medical compression bandages were used in the ICU and medical compression stockings in IMC and regular wards until discharge. The standard pharmacological thrombosis prophylaxis consists of 40 mg/d subcutaneous enoxaparin in the ICU and 4500 IE tinzaparin every 24 h in the IMC. Intravenous and intraarterial catheters were removed as soon as possible at the discretion of the treating physician in the ICU or IMC.^24^ Patients with an indication for therapeutic anticoagulation, for example, atrial fibrillation or thromboembolism, received unfractionated heparin or enoxaparin in therapeutic doses. This regimen was initiated in the ICU as soon as possible after ruling out postoperative active bleeding. Following current guidelines, patients discharged from the hospital did not receive further thrombosis prophylaxis, except for patients who had developed thromboembolism receiving anticoagulation therapy or antiplatelet drugs, according to the current guidelines.

### Statistical Analysis

The Kaplan-Meier method was used to estimate overall survival, and the log-rank test was used to compare survival curves of patients with and without PGD2/3. Fisher exact test and Mann-Whitney *U* test were used when appropriate to evaluate the association between different parameters. Univariate and multivariate logistic regression models were used to select independent predictors of survival. All tests were 2-tailed. A *P* < 0.05 was considered statistically significant. All statistical analyses were conducted using SPSS software (Version 27; IBM Corporation, New York, NY) and GraphPad Prism (Version 9; GraphPad Software, San Diego, CA).

## RESULTS

Overall, 251 patients (133 male, 118 female) underwent LTx at our institution between March 2003 and June 2023. One hundred seventy-three of 251 (69%) of all lung transplant recipients were tested for CAs, which were positive in 78 of 173 (45%) of the patients. One patient underwent preoperative plasmapheresis and was excluded from further statistical analysis. Only 10 patients had a positive direct agglutinin test. Relevant patient characteristics are summarized in Table 1.

**TABLE 1.** Basic patient characteristics

Variable	Recipients, all (n = 173)	Recipients, CA (n = 78)	Recipients, no CA (n = 95)	*P*
Sex				0.45
Male	92 (53%)	44 (56%)	48 (51%)	
Female	81 (47%)	34 (44%)	47 (49%)	
Age at LTX, y				0.25
Minimum	18	22	18	
Maximum	70	68	70	
Median	59	58	60	
Pre-LAS Aera	37 (21%)	22 (28%)	15 (16%)	0.06
LAS Aera	136 (79%)	56 (72%)	80 (84%)	
Minimum	31	32	31	
Maximum	95	95	95	
Median	40	42	41	
Comorbidity				
CHD	31 (18%)		17 (18%)	>0.99
aHT	47 (27%)	19 (24%)	28 (30%)	0.50
Diabetes	36 (21%)	10 (13%)	16 (17%)	0.53
Median PAP mean, mm Hg	26	26	24	0.29
Median BMI	23	22	24	0.08
Pulmonary disease				
COPD	61 (35%)	21 (27%)	40 (42%)	**0.04**
IPF	65 (38%)	35 (45%)	30 (32%)	0.08
CF	9 (5%)	6 (8%)	3 (3%)	0.30
EAA	8 (5%)	2 (3%)	6 (6%)	0.30
AAT	7 (4%)	2 (3%)	5 (5%)	0.46
AI	12 (7%)	5 (6%)	7 (7%)	>0.99
Other	11 (6%)	7 (9%)	4 (4%)	0.23
Operative				
ECMO	57 (33%)	27 (35%)	30 (32%)	0.75
SLTX	21 (12%)	6 (8%)	15 (16%)	0.16
>6 Red cell concentrates	17 (10%)	6 (8%)	11 (12%)	0.45
Median ischemia times, min	432	426	440	0.13
Donor organ quality				
MV, d				
Minimum	1	1	1	0.54
Maximum	19	18	19	
Median	3	3	4	
Po_2_, mm Hg				
Minimum	194	292	194	0.48
Maximum	639	602	639	
Median	434	444	428	
Nicotine	60 (35%)	29 (37%)	31 (33%)	0.63
Donor age				
Minimum	12	16	12	0.43
Maximum	79	79	78	
Median	52	51	53	

Patient characteristics in patients with and without cold agglutinins. Bold entries are statistically significant (*P* < 0.05).

AAT, alpha-1 antitrypsin deficiency; aHT, arterial hypertension; AI, autoimmune disease; BMI, body mass index; CAs, cold agglutinins; CF, cystic fibrosis; CHD, coronary heart disease; COPD, chronic obstructive pulmonary disease; EAA, extrinsic allergic alveolitis; ECMO, extracorporeal membrane oxygenation; IPF, idiopathic pulmonary fibrosis; LAS, lung allocation score; LTx, lung transplantation; MV, mechanical ventilation; PAP, pulmonary artery pressure; Po_2_, Pao_2_ at FiO_2_ 100%; SLTX, single lung transplantation.

**FIGURE 1.**
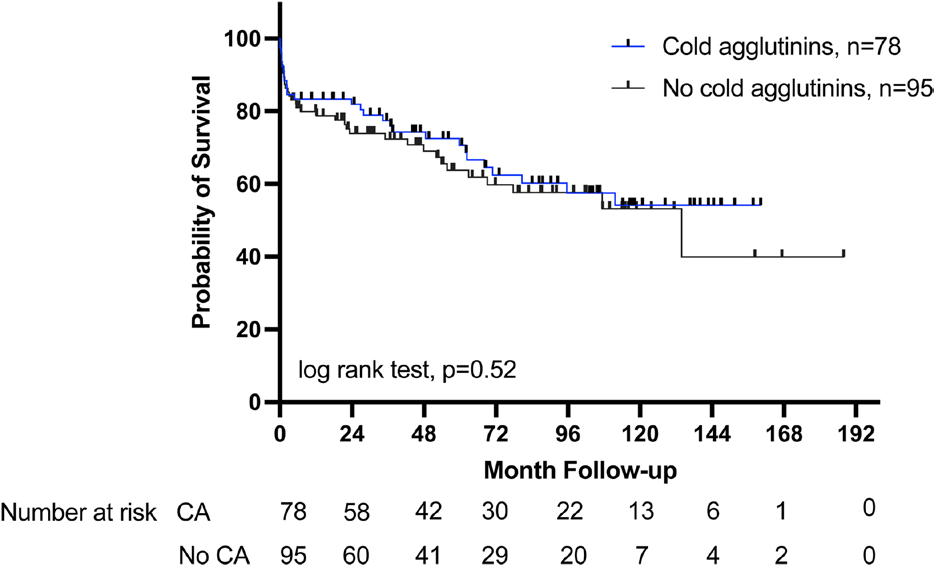
Kaplan-Meier analysis of patients with and without CAs detected before lung transplantation. CA, cold agglutinin.

**FIGURE 2.**
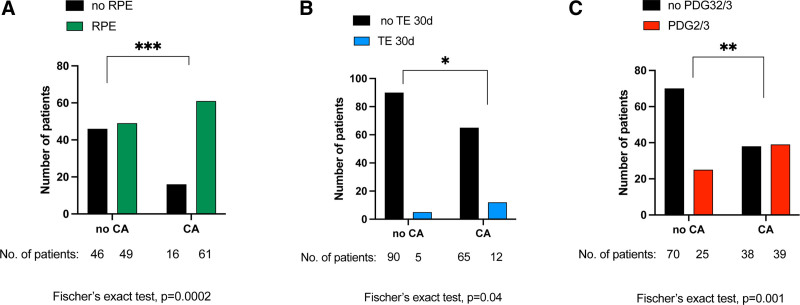
Cold agglutinins are associated with primary graft dysfunction, reperfusion edema, and early postoperative thromboembolism. The presence of CAs associates with RPE (A), early postoperative thromboembolism (B), and severe primary graft dysfunction (C) after lung transplantation. *P ≤ 0.05, **P ≤ 0.01, ***P ≤ 0.001, ****P ≤ 0.0001. CA, cold agglutinins; PGD2/3, primary graft dysfunction grade 2/3; RPE, reperfusion edema; TE 30d, thromboembolism within 30 d after surgery.

There was no difference in 30-d-, 90-d-, or 12-mo mortality and the overall long-term survival in patients with and without CAs (Figure 1). However, the presence of CAs in lung transplant recipients was associated with an increased incidence of reperfusion edema (79% versus 52%; *P* = 00002), severe primary graft dysfunction (PGD2/3; 51% versus 26%; *P* = 0.001), and early postoperative thromboembolism (16% versus 5%; *P* = 0.04; Figure 2) after LTx. The thromboembolic events are further characterized in Table 2.

**TABLE 2.** Early postoperative thromboembolism

Thromboembolism ≤ 30 d	Recipients, all (n = 173)	Recipients, CA (n = 78)	Recipients, no CA (n = 95)	*P*
At least 1 thromboembolic event	17 (10%)	12 (15%)	5 (5%)	**0.04**
>1 thromboembolic event	7 (4%)	5 (6%)	2 (2%)	0.25
Venous thromboembolism	**12 (7%**)	**9 (12%**)	**3 (3%**)	**0.04**
Pulmonary embolism	4 (2%)	3 (4%)	1 (1%)	0.33
Jugular/axillary/subclavian vein thrombosis	8 (5%)	6 (8%)	2 (2%)	0.14
Arterial thromboembolism	**10 (6%**)	**7 (9%**)	**3 (3%**)	0.19
Stroke	8 (5%)	6 (8%)	2 (2%)	0.14
Myocardial infarction	1 (1 %)	0 (0%)	1 (1%)	>0.99
Intestinal ischemia	1 (1%)	1 (1%)	0 (0%)	>0.99

Analysis of thromboembolic events after lung transplantation in patients with and without CAs. Bold entries are statistically significant (*P* < 0.05).

CA, cold agglutinin.

Recipients with chronic obstructive pulmonary disease (COPD) had a lower incidence of CAs (COPD: 27% versus no COPD 44%; *P* = 0.04). While the incidence of CAs was higher in recipients with idiopathic lung fibrosis compared with other pulmonary diseases leading to LTx, this did not reach statistical significance (idiopathic pulmonary fibrosis [IPF]: 54% versus no IPF: 40%; *P* = 0.08). Moreover, there was no significantly increased incidence of chronic lung allograft dysfunction (CLAD) in patients with CAs compared with patients without (14% versus 8%; *P* = 0.23).

In the multivariate analysis, PGD2/3, thromboembolism within the first 30 d, the use of extracorporeal membrane oxygenation (ECMO) and CLAD were independent predictors of reduced survival (*P* = 0.003, *P* = 0.003, *P* = 0.01, and *P* = 0.001, respectively; Table 3). Notably, the number of patients on ECMO during LTX was similar in both groups, with and without CAs (35% versus 32%; *P* = 0.75). There was also no significant difference between both groups’ use of blood products and median ischemia times or other relevant contributors to PGD (Table 1).

**TABLE 3.** Univariate and multivariate Cox regression analyses to identify predictors of survival

Variables (n = 173)	Univariate, HR (95% CI)	*P*	Multivariate, HR (95% CI)	*P*
Perioperative				
Single lung	1.11 (0.49-2.20)	0.78		
ECMO	2.51 (1.52-4.13)	**0.0003**	2.18 (1.20-3.99)	**0.01**
Reperfusion edema	0.53 (0.29-0.91)	**0.03**	0.80 (0.38-1.70)	0.57
PGD2/3	2.39 (1.45-3.93)	**0.0006**	2.68 (1.42-5.32)	**0.003**
Thromboembolism <30 d	2.83 (1.39-5.25)	**0.002**	2.86 (1.37-5.53)	**0.003**
CLAD	2.3 (1.40-3.82)	**0.001**	1.69 (0.90-3.20)	0.10
Recipient characteristics				
Sex, female	0.63 (0.38-1.03)	0.07		
CHD	1.05 (0.52-1.95)	0.89		
Arterial hypertension	0.98 (0.55-1.68)	0.95		
Cold agglutinins	0.85 (0.51-1.40)	0.52		
Autoimmune disease	0.99 (0.44-2.02)	0.99		
mPAP ≥ 25 mm Hg	1.19 (0.72-1.95)	0.49		

Predictors of survival. Bold entries are statistically significant (*P* < 0.05).

CHD, coronary heart disease; CI, confidence interval; CLAD, chronic lung allograft dysfunction; ECMO, extracorporeal membrane oxygenation; HR, hazard ratio; mPAP, mean pulmonary artery pressure; PGD2/3, primary graft dysfunction grade 2/3.

In most patients, CAs were found to be only active at 4 °C. However, 7 of 78 (9%) patients had a temperature amplitude of 22 °C and 2 of 78 (3%) had a temperature amplitude of 37 °C. One of the latter patients died immediately after LTx because of postoperative right heart failure and subsequent multiple organ failure, and the other had a very prolonged postoperative course with multiple thromboembolisms and precipitations being directly visible in blood samples at room temperature (Table 4).

**TABLE 4.** Patients with CA and increased temperature amplitude

Patient	LAS	Lung disease	Sex	Age	BMI	CA	Thermal amplitude (°C)	Reperfusion edema	PGD2/3	Donor age	Donor Pao_2_ at 100%	Donor ventilation (d)	Donor nicotine	Ischemia time (min)	EC	FFP	TC	ECMO	Thromboembolism 30 d	CLAD	Death	Cause of death	Survival (mo)	ICU stay (d)
1	38.6	IPF	Male	48	28	1	22	Yes	No	54	301	11	No	6.22	2	3	0	Yes	0	No	Yes	COVID	62	4
2	Urgent	COPD	Male	55	19	1	22	Yes	No	22	509	2	Yes	NA	NA	NA	NA	NA	0	Yes	Yes	Sepsis	71	3
3	38.6	IPF	Female	66	28	1	37	Yes	Yes	73	386	3	No	7.40	6	5	2	Yes	Stroke, PE, thrombosis	No	Yes	COVID	28	58
4	Urgent	CF	Male	24	16	1	37	Yes	Yes	25	480	11	Yes	NA	13	10	6	Yes	0	NA	Yes	Right heart failure	0.03	<1
5	49.3	GvHD	Male	30	19	1	22	No	No	58	577	5	No	6.17	5	4	2	Yes	0	No	No		21	5
6	33.6	COPD	Female	65	20	1	22	No	No	35	488	9	No	3.15	0	0	0	No	0	No	No		86	1
7	36.2	AAT	Male	51	26	1	22	No	No	16	447	8	No	4.24	0	0	0	No	Stroke	No	No		62	9
8	38.0	CF	Female	55	19	1	22	Yes	Yes	63	530	2	No	4.24	0	0	0	No	Intestinal ischemia, thrombosis	NA	Yes	Intestinal ischemia	0.6	17
9	33.7	COPD	Female	55	19	1	22	Yes	No	35	430	12	No	5.27	0	0	0	No	0	Yes	No		117	2

Patient characteristics of the 9 patients with cold agglutinins and increased temperature amplitude.

AAT, alpha-1 antitrypsin deficiency; BMI, body mass index; CAs, cold agglutinins; CF, cystic fibrosis; CLAD, chronic lung allograft dysfunction; COPD, chronic obstructive pulmonary disease; EC, erythrocyte concentrates; ECMO, extracorporeal membrane oxygenation; FFP, fresh frozen plasma; GvHD, graft-vs-host disease; ICU, intensive care unit; IPF, idiopathic pulmonary fibrosis; LAS, lung allocation score; NA, not available; PE, pulmonary embolism; PGD2/3, primary graft dysfunction grade 2/3; TC, thrombocyte concentrates.

Because of the above-described experience in our center, 2 patients with a high CA titer and broad temperature amplitude underwent plasmapheresis shortly before LTx (the one within the study period, and another patient several months later). This removed the CAs for at least 2 mo with good patient outcomes.

## DISCUSSION

### Incidence and Impact of CAs

To our knowledge, this is the first study analyzing whether the presence of CAs impacts short-term and long-term outcomes in LTx. Data on CAs in other solid organ transplants are limited. While there is no benefit regarding overall long-term survival in LTX recipients with CAs, our data show an increased incidence of reperfusion edema, PGD2/3 and early postoperative thromboembolic events, indicating the potential relevance of previously asymptomatic CAs in our cohort.

In cardiac surgery, asymptomatic patients with CAs can safely undergo normothermic cardiothoracic surgery without further testing. However, in hypothermic conditions, even patients without any symptoms can be at risk for agglutination and hemolysis.^17^ Therefore, in case of higher CA titers or a greater thermal amplitude, a change in management is often recommended.^17^ In clinical practice, the thermal amplitude is often considered more important than the plasma antibody titer.^11^

The reported incidence of CAs among screened cardiac surgical patients is relatively low (approximately 0.8%–4%).^11,25,26^ Surprisingly, the incidence in our study is much higher, with 78 of 173 (45%) of the lung transplant patients testing positive for CAs before transplantation.

While this retrospective study cannot fully answer the question, the higher prevalence of CAs might be related to the underlying pulmonary disease. In the literature, some case reports link the presence of CAs to autoimmune diseases and fibrotic lung disease.^9,10^ Moreover, in the literature, there is a high prevalence of *M. pneumoniae* in COPD patients,^27,28^ an infection associated with the presence of CAs. While some COPD patients in our cohort have a history of infection with *M. pneumoniae*, we do not have sufficient data available to conclude here. We found the percentage of patients with COPD and CAs significantly lower than those without CAs. Correspondingly, there was a higher prevalence of CAs in patients with IPF compared with the rest of the cohort. However, this did not reach statistical significance. Since the rest of the cohort is heterogeneous, more studies are needed to evaluate whether fibrotic lung disease increases the risk of CAs.

In the literature, severe PGD is associated with reduced survival and an increased incidence of CLAD.^29^ In our cohort, we did not find a difference in survival in patients with CAs, and while the incidence of CLAD seemed to be increased in these patients, this did not reach statistical significance. The reason might be small numbers or the fact that the PGD2/3 and CLAD are multifactorial, and one may hypothesize that the influence of CAs may vary depending on titer and temperature amplitude.

Most of our patients had a CA temperature amplitude of only 4 °C. In these patients, we did not put ice in the thoracic cavity before implantation of the respective lungs. Moreover, increasing data support a donor lung preservation strategy at 10 °C.^30-32^ Recent data from Van Slambrouck et al^33^ show that rapid organ rewarming is accompanied by transcriptomic and metabolomic changes in the transplanted lungs, indicating proinflammatory signaling and disturbed cell metabolism. Limiting cooling of the lung might thus alleviate rewarming ischemic injury and might also benefit recipients with CAs.

A broader temperature amplitude was only detected in 9 (12%) patients. Therefore, because of the small numbers, we cannot conclude if the risk for thromboembolic events and PGD2/3 is higher in these patients. The prothrombogenic effect might be general, as suggested by the association of CAs with an increased incidence of thromboembolic events,^7^ possibly pronounced by the transplantation procedure.

Although the etiology of PGD2/3 and thromboembolism is multifactorial, CAs may contribute to it in selected patients. Corroborating this hypothesis, Venkataraman et al^15^ reported a case of a lung donor with known CAD whose lungs were evaluated using ex vivo lung perfusion. The lungs did not flush well during cold preservation, and pulmonary edema developed soon after. Histological evaluation showed agglutinated red blood cells in the microvasculature in pre- and post-ex vivo lung perfusion biopsies, which may have contributed to inadequate parenchymal preservation.^15^ In LTx, there is generally no need for hypothermia. However, the lungs are still cold during the implant procedure. Occasionally, vascular anastomoses require small correctional stitches after reperfusion, leaving blood in an organ with a temperature that may very well be lower than 20 °C. This is speculation at this point, and whether the transplanted lungs are sufficiently hypothermic at the time of reperfusion could be made more probable by testing. However, one might hypothesize that in such a situation, agglutination and endothelial activation may occur in selected patients, and clots form in the lungs’ microcirculation, which might contribute to the development of reperfusion edema and primary graft dysfunction.

Notably, preoperative plasmapheresis was performed immediately before LTx in 3 patients (1 patient in our cohort and 2 who underwent LTX later) with CA activity >22 °C and a high titer removed the CAs for at least 2 mo with good patient outcomes. None of these patients suffered from primary graft dysfunction or early postoperative thromboembolism. Whether this was coincidence or plasmapheresis was indeed beneficial here cannot be answered in the current study.

## LIMITATIONS OF THE STUDY

A limiting factor of this retrospective single-center study is that it covers nearly 20 y. Routine screening for CAs was performed in only 174 of 251 patients, and the laboratory testing standard may have varied over time. Moreover, the etiology of reperfusion edema, primary graft dysfunction and thromboembolism after LTx is multifactorial. This retrospective study cannot fully answer the extent to which CAs contribute to any of these postoperative complications. However, ischemia times, the transfusion of blood products, and the use of ECMO were not significantly different in patients with and without CAs. Yet, because of the small number of patients, part of the statistical results should be seen as descriptive and do not allow for a definite conclusion. Lastly, the activation of CAs depends on temperature amplitude and titer, as well as the temperature of the transplanted organ, which may vary. However, exact details are currently not available for our cohort.

## CONCLUSIONS

There is quite a high prevalence of asymptomatic CA in patients undergoing LTx. While there was no effect on overall long-term survival, we detected an increased incidence of reperfusion edema, severe PGD2/3 and early postoperative thromboembolism in these patients. However, the exact extent of the contribution of CAs to these postoperative complications remains to be further elucidated. Nevertheless, identifying otherwise asymptomatic CAs could allow the multidisciplinary team to change the perioperative management of LTX recipients with high plasma titer and broader temperature amplitude. Further studies are warranted to evaluate whether regular screening benefits selected patients.
